# Use of erectile dysfunction treatments after prostate cancer treatment and their perceived impact on men’s sex life: an analysis of patient reported outcome survey data

**DOI:** 10.1186/s12894-025-01702-0

**Published:** 2025-01-31

**Authors:** Megan Charlick, Tenaw Tiruye, Kerry Ettridge, Michael O’Callaghan, Alex Jay, Kerri Beckmann

**Affiliations:** 1 Cancer Epidemiology and Population Health, University of South Australia, Adelaide, South Australia Australia; 2 Health Policy Centre, South Australian Health and Medical Research Institute, Adelaide, South Australia Australia; 3 South Australian Prostate Cancer Clinical Outcomes Collaborative, Flinders Medical Centre, Flinders University, University of Adelaide, Adelaide, South Australia Australia; 4 Urology Department, Flinders Medical Centre, Bedford Park, South Australia Australia

**Keywords:** Erectile dysfunction, Male, Prostate cancer, Prostatic neoplasm, Sexual function

## Abstract

**Background:**

Although sexual dysfunction is a common treatment side-effect affecting men’s quality of life, many prostate cancer patients do not receive or seek out treatments for erectile dysfunction (ED). The aims of this study are to investigate the extent and patterns of use of ED treatments and their perceived impact at different times following prostate cancer treatment.

**Methods:**

This retrospective cohort study included all men on the South Australian prostate cancer registry who completed one or more Patient Reported Outcome Measures (PROMs) survey from 2016 to 2023 (*n* = 5561). Outcomes included self-reported use of ED treatment (oral medications, intra-cavernosal injections (ICI) and vacuum pumps) and their impact men’s sex life at various time points after treatment. The type and timing of ED treatments used was analysed descriptively. Sociodemographic and clinical characteristics associated with utilisation and self-reported satisfaction were examined using multivariable mixed-effects binomial logistic regression.

**Results:**

Post-treatment use of ED treatments did not exceed 43% at any timepoint, with utilisation rates decreasing over time. Oral medications were most frequently used, while vacuum pump and ICI use was limited. Oral medications were more likely to be used at three-months (odds ratio [OR] = 2.48; 95% confidence interval [95%CI] = 1.88–3.27) and six-months (OR = 2.10; 95%CI = 1.63–2.27) than at 12-months post-treatment, and among men from higher socioeconomic areas (OR = 2.41; 95%CI = 1.47–3.93, highest vs. lowest quintile), and following prostatectomy (OR = 4.37; 95%CI = 2.92–6.42), and less likely among older men (OR = 0.08; 95%CI = 0.05–0.13, < 60yrs vs. 70-79yrs). Men were more likely to report an improved sex life with oral medication use at two-years (OR = 3.79; 95%CI = 1.69–8.47) and five-years (OR = 3.07; 95%CI = 1.51–6.25) post-treatment compared with 12-months or if they were socioeconomically advantaged (OR = 3.22; 95%CI = 1.30–7.96, highest vs. lowest quintile).

**Conclusions:**

A substantial proportion of Australian men do not access or continue to use ED treatments after prostate cancer treatment, with many users reporting only modest effects on their sex life. There is a need to improve access to and maintenance of ED treatments following prostate cancer treatment.

**Supplementary Information:**

The online version contains supplementary material available at 10.1186/s12894-025-01702-0.

## Background

Prostate cancer (PCa) is the most common cancer in Australian men, affecting 1 in 8 men over their lifetime [1]. Treatment options include surgery, radiation therapy, and hormone therapy, all of which negatively impact sexual function [2]. Sexual dysfunction often does not return to pre-treatment levels, with up to 83% of men experiencing erectile dysfunction 15 years post-treatment [3]. Poor sexual function after PCa treatment is associated with lower quality of life, worse mental health, and low masculine self-esteem [4]. Over 50% of men with PCa report unmet sexual health needs [5], which can persist up to 15 years post-diagnosis [6].

Australia health care services comprise both public and private health care providers. All Australian citizens have universal health insurance and free access to the public hospital system. Those with private health insurance can elect to receive care through private providers. Currently around 60% of men with PCa receive primary treatment through the private sector [7]. In Australia, there are no guidelines for penile rehabilitation or the management of sexual dysfunction following PCa treatment, and practices vary by individual clinician and/or clinics. Specialist prostate cancer nurses, who are available in some hospitals are generally responsible for education and support around sexual health needs of men with prostate cancer.

Both the American Urological Association [8] and European Association of Urology [9] have developed guidelines for the management of erectile dysfunction (ED), though neither of these are specific to men treated for PCa. In cases where ED treatments are offered to PCa patients with sexual dysfunction, this usually begins with oral medications (e.g., oral phosphodiesterase-5 inhibitors), and progresses to more invasive treatments including vacuum pumps/erection devices, intracavernosal injections (ICI), and penile implants/prostheses. Each treatment has its disadvantages, primarily their cost, efficacy, and physical side-effects [10]. Typically, oral medications are the recommended first-line therapy but require a degree of intact nerve functioning to be effective [10]. Second-line treatments include vacuum pumps and ICI, both of which are more invasive but are effective independent of nerve sparing status [10].

Prevalence of ED treatment use following prostate cancer is reported to be between 50 and 70% [11, 12]. However, their use is often not sustained long-term, typically due to low rates of satisfaction, the natural return of function, treatment side effects, and financial cost [12–14]. Furthermore, previous studies have rarely explored men’s self-reported perceptions of the impact of ED treatments on their sex life following prostate cancer treatment.

Given the lack of formal guidelines in Australia and the ad hoc nature of support for sexual rehabilitation, this study sought to investigate the use of ED treatments in men with prostate cancer. Using routinely collected PROMs survey data collected by the South Australian Prostate Cancer Clinical Outcomes Collaborative (SA-PCCOC) registry, we aimed to determine the rate of sexual aid use among PCa survivors across different survey time points and to document the self-reported impact of ED treatment use on men’s sex life. As a first step to determining and addressing disparities in sexual health care, we also aimed to identify clinical and socio-demographic factors which were associated with the use of ED treatments and with their perceived impact on men’s sex life.

## Methods

### Data source and sample

This study included 5,561 men from the South Australian Prostate Cancer Clinical Outcomes Collaborative (SA-PCCOC) registry [15] who had completed one or more patient-reported outcome measures (PROMs) surveys between July 2016 and August 2023. No exclusion criteria were applied. SA-PCCOC was established in 1998 as a multisite clinical registry enrolling men diagnosed with prostate cancer in South Australia (SA) treated across both the public and private sector (i.e., 19 of 26 urologists/urology centres and 2/2 radiation oncology providers for most of the study period).

Data on clinical characteristics, treatments and oncological outcomes, as well as PROMs, are collected prospectively. Since 2015, SA-PCCOC has been a contributor to the Australian and New Zealand Prostate Cancer Outcomes registry and captures approximately 80% of prostate cancer cases in SA. During the study period, SA-PCCOC collected PROMs via postal surveys at baseline (pre-treatment), and at 3, 6, 12 and 24 months (until 2020), and 5 years post-treatment. Men on AS or ADT alone, however, do not receive surveys at 3 or 6 months. Response rates across survey time points were approximately 50–60%. However, reaching men prior to initiating treatment was often difficult due to the timing of recruitment to the registry, hence a large proportion of men were not able to be surveyed at baseline. Due to resource limitations, the registry makes no further attempt to remind non-respondents. PROMs surveys included an assessment of men’s physical functioning using the Expanded Prostate Cancer Index Composite (EPIC-26) [16] as well as questions about men’s use of and their self-reported satisfaction with ED treatments, based on questions developed by Schover et al. 2002 [17].

The primary outcomes considered in this study were (1) men’s use of ED treatments and (2) men’s perception of the impact that ED treatment had on their sex life. Men were asked whether they had used any of the following treatments in the past four weeks: (a) Tablets taken by mouth, (b) Injections into the penis, (c) Vacuum devices. For each specific ED treatment used, men were also asked to rate the impact it had on their sex life on a 5-point Likert scale, where 1 = worsened my sex life greatly and 5 = improved my sex life greatly).

Demographic factors (postcode, age at diagnosis), health characteristics (smoking status, body mass index) and clinical and treatment data (diagnostic Gleason score, diagnostic prostate specific antigen (PSA) score, treatment/s received) were also extracted from the registry. Postcodes were used to derive measures of residential remoteness (Accessibility/Remoteness Index of Australia [18]) and relative socio-economic advantage and disadvantage (Australian Bureau of Statistics Socio-Economic Indexes for Australia 2016 [19]). Measures of sexual function and urinary continence (over the past 4 weeks) were derived from EPIC-26, according to recommended scoring conventions for these domains (scores range from 0 to 100, with the later representing the best level of functioning). Self-reported depression (in the past 4 weeks) was derived from a single question in EPIC-26 (item 13c) [20].

### Analysis

Descriptive statistics (frequency distributions and percentages for categorical variables, and medians and interquartile range (IQR) for continuous variables) were used to describe sample characteristics, ED treatments used and self-reported impact on men’s sex life. Chi-squared tests were used to test the difference in use of ED treatments and their impact according to men’s pre-treatment level of sexual function (dichotomised as below (low) or equal to or above (high) the median sexual domain score of 58.3) among the subgroup who had completed baseline and follow-up PROMs surveys.

A series of mixed-effects binomial logistic regression models were conducted to identify factors associated with each of the outcomes of interest: use of each of the ED treatments and their self-reported improvement of ED treatments in men’s sex lives. Covariates included: survey timepoint, treatments prior to survey time point, residential remoteness, relative socio-economic advantage (quintiles ranging from most disadvantaged to most advantaged), age at diagnosis, diagnostic Gleason score, diagnostic PSA level, EPIC-26 continence summary score, current body mass index category, self-reported depression (dichotomised as ‘not at all/very small problem’ vs. ‘small/moderate/big problem’), and smoking status (never, past and current). As there were insufficient numbers of men using pumps or ICI, we were only able to undertake mixed-effects binomial logistic regression models to determine factors associated with self-reported impact of oral medications on men’s sex life (grouped as ‘improved’ = greatly improved/improved and ‘not improved’ = neutral/worsened/greatly worsened). Adjusted odds Ratios (OR), and 95% confidence intervals (CI) are reported throughout. Statistical significance was set at a p-value of 0.05.

Additional subgroup analyses were undertaken among men who underwent radical prostatectomy considering use and self-reported impact at 12 months post-treatment using binary logistic regression models considering the same covariate as above.

Missing data were addressed in two ways: by including an additional ‘missing’ category in final models for variables with a high prevalence of missing values (Gleason score 28.6%, PSA level 8.5%), and via hot deck Imputation for variables with fewer missing values, i.e., < 5% (body mass index, socioeconomic quintiles, depression symptoms and smoking status). Statistical analyses were completed in Stata v18 [21].

## Results

### Sample

In total, 5,561 participants completed 10,387 surveys between 2016 and 2023. The mean age of participants at diagnosis was 67.2 years. Most lived in a major city or inner regional area (83.6%). A large proportion were overweight (46.3%) or obese (27%) and nearly half had undergone radical prostatectomy (46.5%). Median sexual function scores decreased after treatment and did not return to pre-treatment levels. (Table 1)

**Table 1:** Cohort characteristics

Characteristics	*n*	%
**Total number of men**	5561	100
**Age group**		
< 60yrs	894	16.1
60-69yrs	2457	44.2
70-79yrs	1954	35.1
80 + yrs	256	4.6
**Primary treatment^**		
Prostatectomy	2,289	41.2
Radiation therapy	597	24.8
Radiation with ADT	327	5.8
Active Surveillance	909	16.4
Watchful waiting	58	1.0
ADT alone	143	2.6
Chemotherapy	3	0.05
No treatment recorded	1,245	22.4
**Gleason score (total) at diagnosis**		
≤ 6	1,249	22.5
7	1,871	33.6
8–10	851	15.3
Missing	1,590	28.6
**PSA at diagnosis**		
< 4 mg/mL	596	10.7
4– 10 mg/mL	3,165	56.9
> 10 mg/mL	1,327	23.9
Missing	473	8.5
**Place of residence**		
Major City	4,650	83.6
Regional	667	12.0
Remote	244	4.4
**Socio-Economic Index (Quintile)**		
Q1 (Most disadvantaged)	956	17.2
Q2	938	16.9
Q3	849	15.3
Q4	1,096	19.7
Q5 (Most advantaged)	1,705	30.7
Missing	17	0.2
**Body Mass Index Category**		
Underweight - Healthy (< 18–24.99 kg/m^2^)	1,267	22.8
Overweight (25–29.9 kg/m^2^)	2,524	45.4
Obese (> 30 kg/m^2^)	1,499	26.9
Missing	271	4.9
**Smoking status**		
Never smoked	2538	45.7
Past smoker	2597	46.7
Current smoker	385	6.9
Missing	41	0.7
**PROMs timepoints** (***N*** = **10**,**387 surveys)**		
Baseline (pre-treatment)	2,855	27.5
3 months	1,436	13.8
6 months	1,621	15.6
12 months	2,428	23.4
2 years^#^	709	6.8
5 + years	1,338	12.9
**Epic-26 sexual function domain score at**:	**median**	**IQR**
Baseline	58.3	(25.0–83.3)
3 months	16.7	(8.3–34.0)
6 months	16.7	(8.3–36.2)
1 year	22.2	(10.0–57.0)
2 years	22.2	(12.5–57.0)
5 years	22.2	(12.5–54.2)

^ 26% of men on AS transitioned to either RP or XRT

9.3% of men having RP subsequently received ADT or XRT or both during follow-up

Radiation therapy includes low dose rate brachy therapy (*n* = 89)

Radiotherapy plus ADT include men on HDR brachy therapy (*n* = 68)

^#^ PROMs collection at 2-years post treatment ceased from January 2020

At baseline, the overall median sexual function score was 58.3 (IQR 25.0-83.3). At three months, this decreased to 16.7 (IQR 8.3–34.0) and by 12 months had only increased slightly to 22.2 (IQR 10.0–57.0). At baseline (i.e., before treatment), 29% of participants (*n* = 838/2,859) reported their sexual function was a moderate-big problem for them. This increased to 40% (*n* = 648/1,611) at 6 months post-treatment and remained high at 36% (*n* = 491/1,358) five years post-treatment.

### Use of treatments for Erectile Dysfunction

**Fig. 1:**
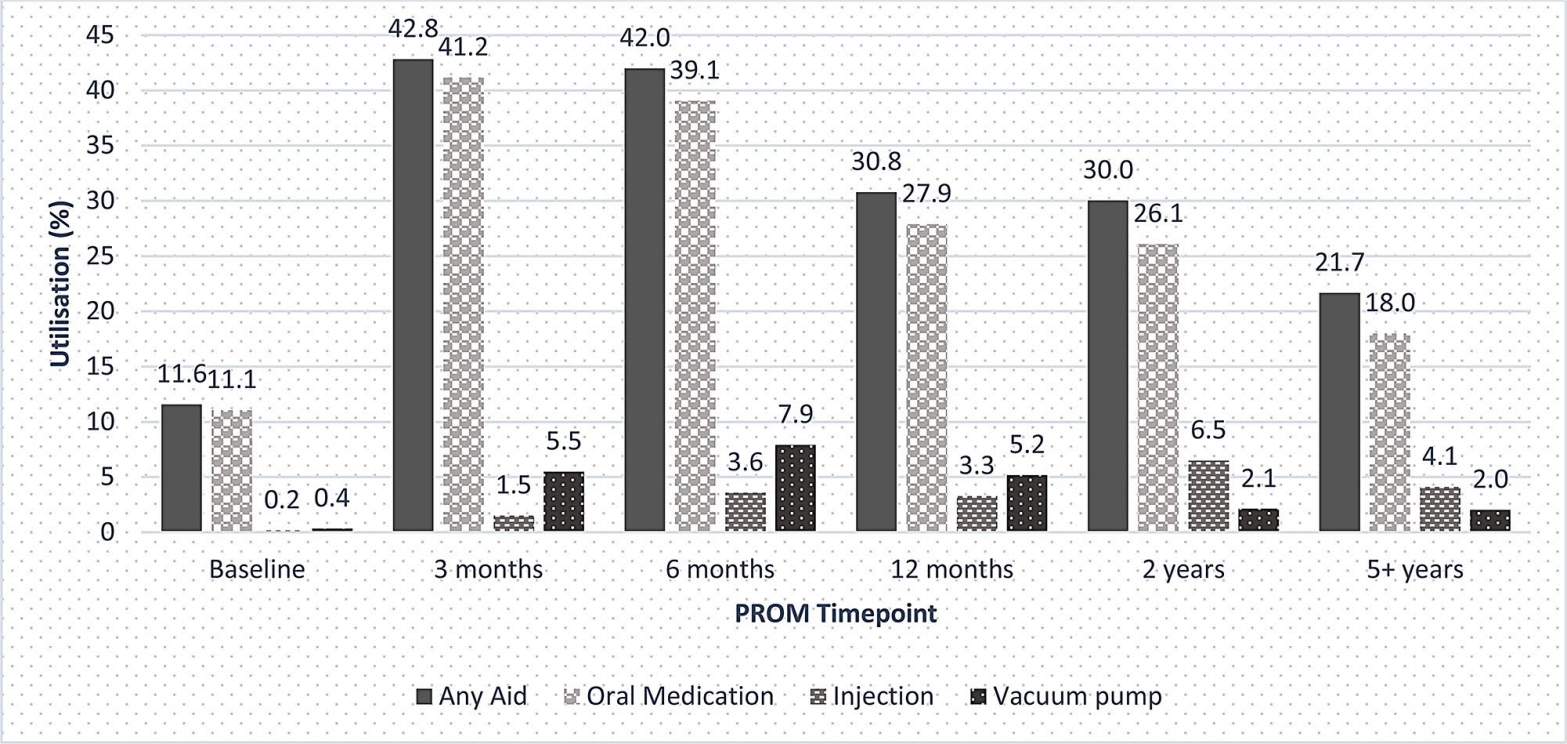
Use of erectile dysfunction treatments over time

Use of ED treatments (in the last four weeks) was highest at three months (42.8%) and lowest at five years (21.7%). Oral medications were the most common type of ED treatment used, followed by vacuum pumps and ICIs. ‘Other’ aids (*n* = 89) included penile implants and penile rings, though most respondents did not specify. Figure 1. Use of ED treatments varied by treatment type but was consistently higher at all time points among men who had undergone radical prostatectomy than those who underwent other types of treatment (Table 2). Among the subgroup with baseline PROMs, use of ED treatments was greater among men who had higher baseline sexual function, with p-values all < 0.001 for oral medications, ICI and vacuum devices (Supplementary Table 1).

**Table 2:** Use of any erectile dysfunction treatments across survey time points by primary treatment approach

Survey time point	Radical prostatectomy		Radiotherapy		Radiotherapy + ADT		Active surveillance*		ADT only*	
	*n*/*N*	%	*n*/*N*	%	*n*/*N*	%	*n*/*N*	%	*n*/*N*	%
Baseline/pretreatment	11/91	12.1	5/39	12.9	0/36	0.0	72/526	13.7	4/123	3.3
3 m	388/782	49.6	18/132	13.6	6/74	8.1	-	-	-	-
6 m	421/835	50.4	21/176	11.9	5/115	6.0	-	-	-	-
12 m	389/840	46.3	42/254	16.5	10/146	7.5	66/363	18.2	3/61	4.9
24 m	132/338	30.1	20/81	24.7	4/31	12.9	14/96	14.6	0/16	0.0
60 m	161/650	25.0	26/153	17.0	5/73	6.9	15/84	17.9	0/9	0.0

n/N = Number of respondents reporting use of any ED treatment in past 4 weeks/Total number within treatment groups completing PROMs at each time point

ADT = androgen deprivation therapy

*Surveys are not administered at 3 and 6 months to men who were managed by active surveillance or ADT alone

Mixed effects logistic regression results indicate that oral medication was more likely to be used in the first six months after treatment (3 months: OR 2.48, 95% CI 1.88–3.27; 6 months: OR 2.10, 95% CI 1.63–2.27), and its use was associated with higher urinary continence scores (OR 1.07, 95% CI 1.01–1.13), having had a radical prostatectomy (OR 4.33, 95% CI 2.92–6.42), being overweight (OR 1.52, 95% CI 1.07–2.15) and being socioeconomically advantaged (Q5 vs. Q1: OR 2.41, 95% CI 1.47–3.93). ICI use was more likely among men who underwent radical prostatectomy (OR 13.76, 95% CI 3.81–49.69), those living in regional areas (OR 2.78, 95% CI 1.05–7.35), and those who were more socioeconomically advantaged (Quintile 4 vs. 1), OR 3.06, 95% CI 1.08–8.68). Men who were older and only 3-months post-treatment were less likely to report using ICI.

For vacuum pump use, mixed effects logistic regression results (reported at 300 maximum iterations due to non-convergence) indicate that men were more likely to report using vacuum pumps if they had undergone radical prostatectomy (OR 17.97, 95% CI 6.55–49.28) and less likely to report using pumps at three months and five years post-diagnosis, and if they had radiation treatment, or were older. (Table 3)

**Table 3:** Factors associated with use of specific erectile dysfunction treatments after prostate cancer treatment/management

Factors		Oral Medications			Intracavernosal Injections			Vacuum Pumps		
		OR	95% CI	*P* value	OR	95% CI	*P* value	OR	95% CI	*P* value
Time post-treatment:	3 months	2.48	1.88–3.27	< 0.001	0.19	0.09–0.40	< 0.001	0.42	0.23–0.78	0.006
	6 months	2.10	1.63–2.27	< 0.001	0.84	0.49–1.44	0.530	1.35	0.80–2.27	0.260
	12 months	1.00	reference	-	1.00	reference	-	1.00	reference	-
	2 years	0.59	0.39–0.87	0.008	1.94	1.00–3.77	0.051	0.38	0.12–1.22	0.104
	5 + years	0.21	0.15–0.30	< 0.001	0.76	0.40–1.42	0.386	0.16	0.06–0.42	< 0.001
Age at diagnosis:	< 60 years	1.00	reference	-	1.00	reference	-	1.00	reference	-
	60–69 years	0.36	0.24–0.54	< 0.001	0.57	0.28–1.16	0.122	0.45	0.19–1.04	0.062
	70–79 years	0.08	0.05–0.13	< 0.001	0.23	0.09–0.58	0.002	0.07	0.02–0.26	< 0.001
	≥ 80 years	0.02	0.00–0.05	< 0.001	0.04	0.00–1.03	0.052			
Treatment received before completing PROMS (yes v no):	Radical prostatectomy	4.33	2.92–6.42	< 0.001	13.8	3.81–49.7	< 0.001	18.0	6.55–49.2	< 0.001
	Radiation therapy	0.49	0.32–0.73	0.001	0.95	0.42–2.15	0.897	0.13	0.03–0.54	0.005
	Hormone therapy	0.26	0.12–0.58	0.001	0.75	0.18–3.13	0.691	0.71	0.08–6.58	0.765
	Observation*	0.69	0.43–1.11	0.125	0.74	0.25–2.22	0.590	0.45	0.10–2.11	0.312
PSA at diagnosis:	<4 ng/mL	1.00	reference	-	1.00	reference	-	1.00	reference	-
	4–10 ng/mL	0.58	0.36–0.92	0.020	0.88	0.33–2.32	0.797	0.82	0.26–2.58	0.732
	> 10 ng/mL	0.28	0.16–0.49	< 0.001	1.07	0.36–3.24	0.9	1.02	0.27–3.78	0.978
Gleason score:	≤ 6	1.00	reference	-	1.00	reference	-	1.00	reference	-
	7	1.48	0.96–2.28	0.076	2.35	0.94–5.89	0.067	2.66	0.58–12.2	0.209
	8–10	0.62	0.35–1.10	0.103	1.81	0.57–5.72	0.312	1.58	0.23–10.8	0.640
Geographic location:	Major city/outer urban	1.00	reference	-	1.00	reference	-	1.00	reference	-
	Outer Regional	0.96	0.58–1.58	0.869	2.78	1.05–7.35	0.040	2.01	0.70–5.83	0.196
	Remote/Very remote	1.33	0.66–2.68	0.426	2.56	0.66–9.90	0.172			
Socioeconomic Status (quintiles):	Most disadvantaged Q1	1.00	reference	-	1.00	reference	-	1.00	reference	-
	Q2	1.45	0.87–2.41	0.152	0.98	0.33–2.92	0.969	1.78	0.48–6.64	0.392
	Q3	1.30	0.76–2.23	0.345	0.73	0.21–2.51	0.620	0.95	0.21–4.33	0.945
	Q4	1.68	1.01–2.80	0.047	3.06	1.08–8.68	0.035	1.58	0.41–6.02	0.504
	Most advantaged Q5	2.41	1.47–3.93	< 0.001	2.39	0.84–6.81	0.104	1.11	0.29–4.30	0.877
Continence score:	per 10 pt score increase	1.07	1.01–1.13	0.020	1.03	0.92–1.15	0.642	0.99	0.87–1.13	0.933
Body mass index:	Healthy	1.00	reference	-	1.00	reference	-	1.00	reference	-
	Overweight	1.52	1.07–2.15	0.018	0.83	0.41–1.68	0.613	1.33	0.51–3.44	0.558
	Obese	0.70	0.47–1.05	0.084	0.85	0.43–2.07	0.889	0.95	0.32–2.85	0.929
Smoking status:	Never smoked	1.00	-	-	1.00	reference	-	1.00	reference	-
	Past smoker	0.77	0.58–1.01	0.061	0.89	0.50–1.57	0.684	0.74	0.36–1.52	0.415
	Current smoker	0.68	0.38–1.21	0.191	1.89	0.68–5.26	0.224	0.36	0.06–2.10	0.256
Depression symptoms	Small-large *v* none/very small problem	0.48	0.27–0.84	0.011	0.52	0.15–1.76	0.290	2.00	0.52–7.68	0.313

*Observation includes Active surveillance (95%) and watchful waiting (5%)

### Self-reported impact of Erectile Dysfunction treatments

Overall, most men perceived the impact of ED treatments on their sex life to either be neutral or positive, with few men reporting a negative impact. However, perceptions differed by the type of ED treatment and tended to fluctuate over time. At three and six months after PCa treatment most men reported that oral medication use had no impact on their sex life (72% and 62.7%, respectively), while at two- and five- years post treatment the majority reported that they improved their sex life (64.2% and 65.3%, respectively). In comparison, the majority of men who used ICIs reported that these aids improved their sex life at all time points (Table 4). Among the subset of men who had completed baseline and follow-up PROMs surveys, more men with high baseline sexual function than low sexual function reported improvement in their sex life with oral medication (37.5% v 29.1%, *p* = 0.007) and injection (77.5% vs. 55.1%, *p* = 0.013) use (Supplementary Table 1).

**Table 4:** Self-reported perceived impact of sexual aids on sex life over time

PROM timepoint	Greatly worsened/ Worsened *n* (%)	Neither *n* (%)	Greatly improved/ Improved *n* (%)	Total *N*
**Oral Medications**				
Baseline	7 (2.2%)	65 (20.8%)	241 (77.0%)	313
3 Months	31 (5.3%)	422 (72.0%)	133 (22.7%)	586
6 Months	29 (4.6%)	391 (62.6%)	205 (32.8%)	625
12 Months	33 (4.9%)	304 (45.3%)	334 (49.8%)	671
2 Years	6 (3.3%)	57 (31.3%)	119 (65.4%)	182
5 + Years	10 (4.2%)	72 (30.1%)	157 (65.7%)	239
**Injections**				
Baseline	1 (25.0%)	0	3 (75%)	4
3 Months	3 (13.6%)	6 (27.3%)	13 (59.1%)	22
6 Months	2 (3.6%)	11 (19.6%)	43 (76.8%)	56
12 Months	9 (11.3%)	19 (23.8%)	52 (65%)	80
2 Years	4 (8.7%)	7 (15.2%)	35 (76.1%)	46
5 + Years	2 (3.7%)	10 (18.5%)	42 (77.8%)	54
**Vacuum Pump**				
Baseline	1 (11.1%)	3 (33.3%)	5 (55.6%)	9
3 Months	5 (6.4%)	47 (60.3%)	26 (33.3%)	78
6 Months	7 (5.5%)	74 (58.3%)	46 (36.2%)	127
12 Months	6 (4.9%)	63 (51.6%)	53 (43.4%)	122
2 Years	1 (6.7%)	6 (40%)	8 (53.3%)	15
5 Years	4 (14.8%)	10 (37.0%)	13 (48.2%)	27

**Table 5:** Factors associated with self-reported improvement in sex-life with use of oral medications for erectile dysfunction

Factors		Oral medication users (*n* = 1,394 men)		
		OR	95% CI	*p*-value
Time post-treatment:	3 months	0.14	0.08–0.23	< 0.001
	6 months	0.28	0.18–0.44	< 0.001
	12 months	1.00	reference	-
	2 years	3.79	1.69–8.47	0.001
	5 + years	3.07	1.51–1.56	0.002
Age at diagnosis:	< 60 years	1.00	reference	-
	60–69 years	0.35	0.19–0.65	0.001
	70–79 years	0.32	0.15–0.68	0.003
	≥ 80 years	0.48	0.04–6.44	0.577
Treatment received before completing PROMS (yes v no):	Radical prostatectomy	0.36	0.18–0.71	0.004
	Radiation therapy	1.16	0.49–2.79	0.734
	Hormone therapy	1.22	0.20–7.30	0.830
	Observation*	2.01	0.84–4.79	0.117
PSA at diagnosis:	<4 ng/mL	1.00	reference	-
	4–10 ng/mL	1.19	0.56–2.52	0.649
	> 10 ng/mL	0.61	0.24–1.56	0.296
Gleason score:	≤ 6	1.00	reference	-
	7	0.45	0.21–0.97	0.043
	8–10	0.09	0.03–0.26	< 0.001
Geographic location:	Major city/outer urban	1.00	reference	-
	Outer Regional	1.77	0.70–4.45	0.228
	Remote/Very remote	1.30	0.38–4.50	0.117
Socioeconomic Status (quintiles):	Most disadvantaged Q1	1.00	reference	-
	Q2	0.79	0.31–1.98	0.614
	Q3	1.15	0.42–3.16	0.790
	Q4	0.97	0.38–2.47	0.957
	Most advantaged Q5	3.22	1.30–7.96	0.011
Continence score:	per 10 pt score increase	1.40		
Body mass index:	Healthy	1.00	reference	-
	Overweight	1.16	0.64–2.11	0.634
	Obese	0.67	0.33–1.37	0.270
Smoking status:	Never smoked	1.00	-	-
	Past smoker	0.70	0.43–1.15	0.158
	Current smoker	0.70	0.24–2.23	0.511
Depression symptoms	Small-large *v* none/very small problem	0.66	0.20–2.23	0.508

*Observation includes Active surveillance (95%) and watchful waiting (5%)

Results of mixed effect models (Table 5), also indicated that men were more likely to report that the use of oral medications have improved their sex life at 2-years (OR 3.79, 95% CI 1.69–8.47) and 5-years (OR 3.07, 95% CI 1.51–6.25) post-treatment, relative to 12 months post treatment, but less likely to report a positive impact at three months (OR 0.14, 95% CI 0.08–0.23) and six months (OR 0.28, 95% CI 0.18–0.44) post treatment. Men with better urinary continence scores (OR 1.40, 95% CI 1.25–1.56 per 10 point increase) and those living in high socioeconomic areas (OR 3.22, 95% CI 1.30–7.96, highest vs. lowest SES quintile) were more likely to report that oral medication use improved their sex life, while older men (OR 0.32, 95% CI 0.15–0.68, 70-79yrs vs. < 60yrs), those with higher grade disease (OR 0.09, 95% CI 0.03–0.26, Gleason score 8–10 vs. 6) and those who underwent radical prostatectomy compared to those who had not (OR 0.36, 95%CI 0.18–0.71) were less likely to report an improvement in their sex life. Closer examination of men who had undergone radical prostatectomy indicated younger age, better continence scores and higher socioeconomic status were associated with perceived improvement in men’s sex-life with oral ED medications at 12 months post-prostatectomy. Higher Gleason score and being a current smoker were associated with lower likelihood of reporting a positive impact on their sex-life. (Supplementary Table 2)

## Discussion

This large cohort study of men with PCa found that, despite substantial declines in sexual function after treatment, which generally do not return to pre-treatment levels, the prevalence of sexual aid use to improve sexual function did not exceed 43% at any measured time point (with utilisation rates decreasing with increasing time since diagnosis). Prevalence in our study is slightly lower compared to other literature suggesting that around half of prostate cancer patients will use ED treatments in their post-treatment journey (7,8). Oral medications were the most popular and ICI were the least popular ED treatments used. The perceived impact of ED treatments on sex life was moderate, though this varied by type and across time points. Participants using ICI tended to report the most positive impacts, with fewer participants using oral medications reporting favourable impacts. For oral medications, the greatest effect was reported at two- and five-years post-treatment, and among young and more socioeconomically advantaged men.

Our results indicate declining use of ED treatments over time, despite little improvement in sexual functioning beyond 12 months after PCa treatment. Limited research has investigated PCa patient’s reasons for discontinuing ED treatment, despite high rates of unmet sexual needs [6], though reported factors often include treatment side effects, high financial costs, and dissatisfaction with treatment efficacy [10, 11, 13, 14]. Furthermore, men who undergo PCa treatment are often reported to have overly optimistic expectations regarding their sexual function post-treatment and the efficacy of ED treatments [22, 23], as well as negative attitudes towards artificially assisted sex and an active avoidance in planning for sexual rehabilitation post-treatment [24]. The barriers to the uptake and continued use of ED treatments require further investigation to ensure men’s needs can be adequately and effectively addressed.

Since oral medication is generally recommended as a first line treatment for erectile dysfunction, it is not surprising that it was the most frequently reported sexual aid used by participants in our study. Oral medication use was over four times more likely in men who had a radical prostatectomy. A recent scoping review [25] reported that men treated with radical prostatectomy were more likely to be offered and access support to address sexual function. Several studies have found that men treated with radiotherapy, hormone therapy, or active surveillance were less likely to be asked about erectile function by a healthcare practitioner or offered treatment to address it [26, 27]. In the case of radiotherapy, this difference may be due to a belief that there is less chance of permanent sexual function loss compared to surgical treatments [28]. Because the impact of radiotherapy on sexual function tends to be more gradual compared to surgical treatments [29], radiation oncologists may be less inclined to prioritise sexual recovery during follow-up care. In addition, research suggests that delaying the start of penile rehabilitation after radical prostatectomy is associated with poorer erectile function scores [10, 30], leading to more recommendations and use of medications following radical prostatectomy than after other treatments. However, no formal penile rehabilitation services currently exist in South Australia, though in some practices oral medications are prescribed to men who undergo radical prostatectomy.

Our study also indicated a strong inverse association between use of ED treatments and age. The forementioned scoping review also highlighted older age as a barrier to receiving adequate support around sexual issues after prostate cancer treatment [25]. This may also stem, in part, from an erroneous assumption that older men are not interested in their sexual wellbeing [31]. To ensure adequate follow-up care it is important that healthcare practitioners to regularly initiate conversations with patients, regardless of their age or the treatments they received.

In the present study, oral medication was not generally viewed as being effective in the first six months post-treatment, though the perceived impact was more likely to be positive at two- and five-years post-treatment. Given the decline in prevalence with increasing time post-treatment, these results may reflect ongoing use among men who found oral medication to be effective. For men who underwent radical prostatectomy, the effectiveness of oral medications was limited for men with more severe clinical characteristics (higher Gleason scores) and among current smokers. The association with grade may be due to a higher propensity for non-nerve sparing surgery in these men since higher grade may necessitate more cautious approach to ensure positive oncological outcomes. The negative effects of smoking on sexual functioning post-prostatectomy have been documented in a recent meta-analysis [32], with our data indicating this may extend to lack of effectiveness of oral ED medications.

Very few men in the present study reported using more invasive ED treatments such as ICI and vacuum pumps, despite these options not requiring intact nerves and having high efficacy rates [10, 13]. More participants reported using vacuum pumps compared to ICI, though in both cases they were used in combination with oral medications. Reasons for low uptake and sustained use of ICI are also somewhat under-researched. However, men have reported a general dislike of this treatment (particularly due to needle use), experiencing side effects such as painful erections, fear of rare side effects like priapism, treatment inefficacy, and the natural return of erections with time [33]. One novel intervention designed to increase uptake and continued use of ICI for penile rehabilitation post PCa treatment is the use of acceptance and commitment therapy (ACT) [34]. Preliminary results from this pilot randomised controlled trial of 53 men indicate that participants who receive ACT used more penile ICI per week and were more adherent to the rehabilitation protocol, have greater satisfaction with treatment, greater sexual self-esteem and sexual confidence, lower sexual bother, and lower PCa treatment regret compared to the standard penile rehabilitation group [34].

Limitations of this study include a low response rate to the SA-PCCOC registry PROMs survey (50–60%), and therefore the sample may not fully reflect all patient experiences. There is also a strong possibility that response bias could have impacted our findings, in that men with greater levels of sexual dysfunction may respond more frequently to PROMs surveys. However, PROMs questionnaires assessed a variety of outcomes in addition to sexual outcomes. The self-reported nature of our outcome measures is also a potential limitation since this may have introduced measurement errors and, or biases. Findings relating to the impact of ED treatments on men’s sex lives should not be taken to indicate the extent of physiological improvement in sexual function. However, our findings are still of value since they reflect the benefits men felt regarding their overall sexual wellbeing. Unfortunately, we were unable to perform a true longitudinal analysis, as most participants had only completed two or fewer surveys at the time of analysis, and baseline results were unavailable for large proportion of the sample (due to low ascertainment prior to commencing treatment). Analysis of perceived efficacy of pumps and ICI were not possible due to the small number of men using these treatments. Also, we were not able to distinguish whether men were provided treatments for sexual dysfunction as part of a penile rehabilitation program or sought them on their own. Due to difference across health care settings internationally, generalisability may be limited to the Australian health care context. Even within the Australian health care setting, we expect there is considerable variability, given the lack of guidelines for penile rehabilitation and no common pathway for men seeking sexual health care support after prostate cancer.

In terms of strengths, this is a relatively large sample of Australian men, using prospectively collected clinical and PROMs data from a clinical registry with near population-wide coverage (> 80% of PCa diagnosed in the state). Furthermore, it reports on patient’s subjective experience regarding the impact of ED treatments on their sex-life, which is largely underreported in similar research on the use of ED treatments.

## Conclusions

Findings suggest that a substantial proportion of men do not access ED treatments to improve sexual function after prostate cancer treatment. Use of ED treatments does not appear to be sustained over time and generally their impact is perceived to be modest. Injection use was low across all timepoints despite its reported positive impact on sex life and evidence of its efficacy. Further investigation into the barriers to men accessing ED treatments and potential interventions to support continued use is required for men’s unmet sexual needs to be adequately addressed.

## Electronic supplementary material

Below is the link to the electronic supplementary material.

Supplementary Material 1

## Data Availability

The data that support the evidence included in this research are available from SA-PCCOC registry (https://www.prostatehealth.org.au/) but restrictions apply to the availability of these data, which were used under license for the current study, and so are not publicly available. Data are however available from the authors upon reasonable request and with permission of SA-PCCOC data custodians.
