# Supplementary material for: Use of erectile dysfunction treatments after prostate cancer treatment and their perceived impact on men’s sex life: an analysis of patient reported outcome survey data
Source: BMC Urol. 2025 Jan 31;25:21. doi: 10.1186/s12894-025-01702-0 (PMC11783798; doi:10.1186/s12894-025-01702-0)
Supplement: Supplementary file 1 — Supplementary Material 1 [file 12894_2025_1702_MOESM1_ESM.docx]

**Supplementary Tables**

**Supplementary Table 1. Use of any ED treatment according to level of baseline sexual function**

|  | **Low sexual function** | **High sexual function** |  |
| --- | --- | --- | --- |
| Any post-treatment use of: | n/N (% of responders) | n/N (% of responders) | p-value |
| Oral medication | 322/1321 (24.4) | 807/1548 (52.1) | <0.001 |
| Vacuum devices | 68/1256 (5.4) | 133/1401 (9.5) | <0.001 |
| ICI (injections) | 32/12259 (2.5) | 53/1414 (3.8) | <0.001 |
|  |  |  |  |
| Improvement in sex life: |  |  |  |
| Oral medication | 95/327 (29.1) | 303/809 (37.5) | 0.007 |
| Vacuum device | 22/68 (32.4) | 60/132 (45.5) | 0.074 |
| Injections | 17/33 (55.1) | 41/53 (77.4) | 0.013 |

Footnotes:

Number of men with baseline and follow-up survey data (any post treatment time point) = 2855

Low and high baseline sexual function defined as those below and above the median EPIC26 Sexual function score (58.3)

Odds of oral medications use after prostate cancer treatment among men with high compared with low baseline sexual function (adjusted for other covariates) = 5.93; 95% CI = 3.58-9.81, p<0.001.

Odds of reporting improved sex life with oral medication use among men with high compared with low baseline sexual function (adjusted for other covariates) = 1.75; 95% CI = 0.77-3.96, p<0.178.

**Supplementary Table 2. Factors associated with self-reported improvement in sex life with use of oral ED medications use 12 months after radical prostatectomy.**

| **Factors** | | **Oral Medication**  n=407/969 (40.5%) | | |
| --- | --- | --- | --- | --- |
|  |  | **OR** | **95% CI** | **P value** |
| Age at diagnosis: | <60 years | 1.00 | reference | - |
|  | 60-69 years | 0.47 | 0.27-0.79 | 0.005 |
|  | 70-79 years | 0.44 | 0.22-0.86 | 0.017 |
|  | >80 years | 0.32 | 0.02-5.57 | 0.442 |
| PSA score at diagnosis: | <4 ng/mL | 1.00 | reference | - |
|  | 4 - 10 ng/mL | 1.36 | 0.68-2.72 | 0.381 |
|  | >10 ng/mL | 1.48 | 0.60-3.67 | 0.503 |
| Gleason score | ≤6 | 1.00 | reference | - |
|  | 7 | 0.52 | 0.28-0.94 | 0.030 |
|  | 8-10 | 0.24 | 0.10-0.56 | 0.001 |
| Geographic location: | Major city/Inner regional | 1.00 | reference | - |
|  | Outer regional | 1.11 | 0.43-2.90 | 0.831 |
|  | Remote/very remote | 1.16 | 0.40-3.38 | 0.779 |
| Socio-economic status | Most disadvantaged Q1 | 1.00 | reference | - |
| (quintiles): | Q2 | 1.46 | 0.57-3.79 | 0.428 |
|  | Q3 | 1.49 | 0.51-4.35 | 0.465 |
|  | Q4 | 1.75 | 0.69-4.46 | 0.237 |
|  | Most advantaged Q5 | 1.94 | 0.77-4.89 | 0.775 |
| Urinary incontinence | per 10unit increase in score | 1.24 | 1.11-1.38 | <0.001 |
| Body mass index | Healthy weight <25 | 1.00 | reference | - |
| *kg/m^2^*: | Overweight (25-29.9) | 1.32 | 0.80-2.28 | 0.311 |
|  | Obese (≥30) | 0.93 | 0.48-1.77 | 0.827 |
| Smoking status: | Never smoked | 1.00 | reference | - |
|  | Past smoker | 1.19 | 0.76-1.87 | 0.449 |
|  | Current smoker | 0.34 | 0.12-0.96 | 0.042 |
| Self-report depression | Small-big bother vs none/very small problem | 0.25 | 0.03-2.40 | 0.229 |
